# Exclusivity of breastfeeding and body composition: learnings from the Baby-bod study

**DOI:** 10.1186/s13006-021-00389-x

**Published:** 2021-05-19

**Authors:** Sisitha Jayasinghe, Manoja P. Herath, Jeffrey M. Beckett, Kiran D. K. Ahuja, Nuala M. Byrne, Andrew P. Hills

**Affiliations:** School of Health Sciences, College of Health and Medicine, University of Tasmania, Locked Bag 1322, Newnham Drive, Launceston, TAS 7250 Australia

**Keywords:** Exclusive breastfeeding, Non-exclusive breastfeeding, Body composition, Weight retention, Obesity

## Abstract

**Background:**

This report evaluated the breastfeeding status in a Tasmanian cohort and its effects on infant and maternal anthropometry and body composition.

**Methods:**

An observational-cohort analysis of self-reported feeding data from 175 Tasmanian mother-baby dyads (recruited via in-person contact between September 2017 and October 2019), was executed. Only mothers who were ≥ 18 years of age, who had a singleton pregnancy and were able to speak and understand English, were included in the study. Infants outside a gestational age range between 37^+ 0^ and 41^+ 6^ weeks were excluded. Infant (using Air Displacement Plethysmography) and maternal body composition was assessed at 0, 3 and 6 months. Analysis of variance with relevant statistical corrections were utilised for cross-sectional and longitudinal comparisons between non-exclusively breastfed (neBF) and exclusively breastfed (eBF) groups.

**Results:**

Fat-free mass was significantly higher [*t* = 2.27, *df* = 98, *P* = 0.03, confidence interval (CI) 0.03, 0.48] in neBF infants at 6 months (5.59 ± 0.59 vs 5.33 ± 0.50 kg) despite a higher mean fat-free mass in eBF infants at birth (2.89 ± 0.34 vs 3.01 ± 0.35 kg). Weak evidence for different fat mass index trajectories was observed for eBF and neBF infants in the first 6 months of life (ANOVA, *F* = 2.42, *df* = 1.9, *P* = 0.09) with an inversion in fat mass index levels between 3 and 6 months. Body Mass Index (BMI) trajectories were significantly different in eBF and neBF mothers through pregnancy and the first 6 months postpartum (ANOVA, F = 5.56, df = 30.14, *P* = 0.01). Compared with eBF mothers, neBF mothers retained significantly less weight (t = − 2.754, *df* = 158, *P* = 0.02, CI -6.64, − 1.09) at 3 months (0.68 ± 11.69 vs 4.55 ± 6.08 kg) postpartum. Prevalence for neBF was incrementally higher in mothers with a normal BMI compared to mothers with obesity, and mothers who underwent surgical or medical intervention during birth were less likely to exclusively breastfeed.

**Conclusions:**

Infants with different feeding patterns may display varying growth patterns in early life and sustained breastfeeding can contribute to greater postpartum maternal weight loss.

## Background

Prenatal and early postnatal nutrition plays a significant role in reducing the risk of childhood obesity [1, 2]. In this context, breast milk has been identified as one of the most efficient, natural, cost-effective means of optimising nutrition in early life [3, 4]. Although the exact mechanisms are yet to be elucidated, a substantial body of literature supports the preventative capacity of breastfeeding during infancy on later life incidence of obesity and related comorbidities [5–8]. The benefit to infants from prolonged breast milk consumption is not limited to prevention of obesity; it is also associated with cognitive, immune, and healthy digestive system development [9–11].

Breastfeeding/ lactation can also have a profound impact on maternal health. Evidence indicates that breastfeeding can reduce the incidence of numerous metabolic and physiological complications in mothers, including type 2 diabetes [12, 13], metabolic syndrome [14] and cardiovascular disease [15]. Pregnancy is commonly associated with an increase in visceral fat, insulin production and circulating lipid levels [16], and a delayed return to pre-pregnancy levels may place mothers at an elevated risk of developing deleterious metabolic conditions. According to the ‘Reset Hypothesis’, lactation can reverse some of these trends via mobilization of fat stores accumulated during pregnancy and be a potential vehicle for reductions in the incidence of metabolic disease [17]. Lactation also plays an important role in postpartum maternal weight management with exclusive breastfeeding (eBF) acting as an effective means of weight loss following childbirth [18, 19].

Despite the acknowledged importance of eBF as the best possible nutritional start to life for infants, 78 million babies worldwide (estimated in 2018) are not breastfed within the first hour of life [20]. The ideal timeframe for eBF lacks global consensus; however, the World Health Organization (WHO) recommends eBF for at least 6 months for all infants [21–23]. Initiation, duration, continuity, and cessation of breastfeeding depend on a multitude of factors including maternal obesity, use of medications (particularly ones that are prescribed at labour), perceived insufficient milk supply, and mode of birth/birth complications [24–29]. In addition, socio-economic status is considered to be a major determinant of breastfeeding pattern [30]. Previous reports have suggested that socioeconomically disadvantaged regions in Australia, including in Tasmania, have low rates of breastfeeding compared with other parts of the country [31, 32]. According to the Department of Health and Human Services, 75–85% (as opposed to the recommended 90%) of Tasmanian mothers breastfeed at the time of discharge from hospital following childbirth [33]. Further, the number of women who breastfeed drops over the first 6 months with only 44% of mothers partially breastfeeding (as opposed to the recommended 80%) at 6 months postpartum [33]. This report evaluated breastfeeding trends in a Tasmanian cohort and its effects on both infant and maternal anthropometry and body composition.

## Methods

### Participants

Participant recruitment and data collection was undertaken as part of a larger study (‘Developing better information globally on young children’s body composition’) at the maternity ward of the Launceston General Hospital in the north of Tasmania, Australia. Briefly, mothers admitted to the postnatal ward at Launceston General Hospital were approached by trained research staff and midwives and given a plain language description of the study. Subsequently, subject to the provision of written informed consent, all interested, eligible mothers were enrolled in the study. Only mothers who were ≥ 18 years of age, who had a singleton pregnancy and were able to speak and understand English, were included in the study. Infants outside a gestational age range between 37^+ 0^ and 41^+ 6^ weeks were excluded. Further, women who experienced significant complications at labour (according to the assessment of the attending clinician), and infants born with a congenital anomaly, were also not included. According to self-reported data from consenting mothers, approximately 90% were Caucasian (Tasmanian average 83.5%). Average maternal age was 30.3 years (range 18–48) and infants were 1.7, 87.7 and 180.4 days old (on average) at birth, 3-month and 6-month data collection points, respectively.

**Fig. 1:**
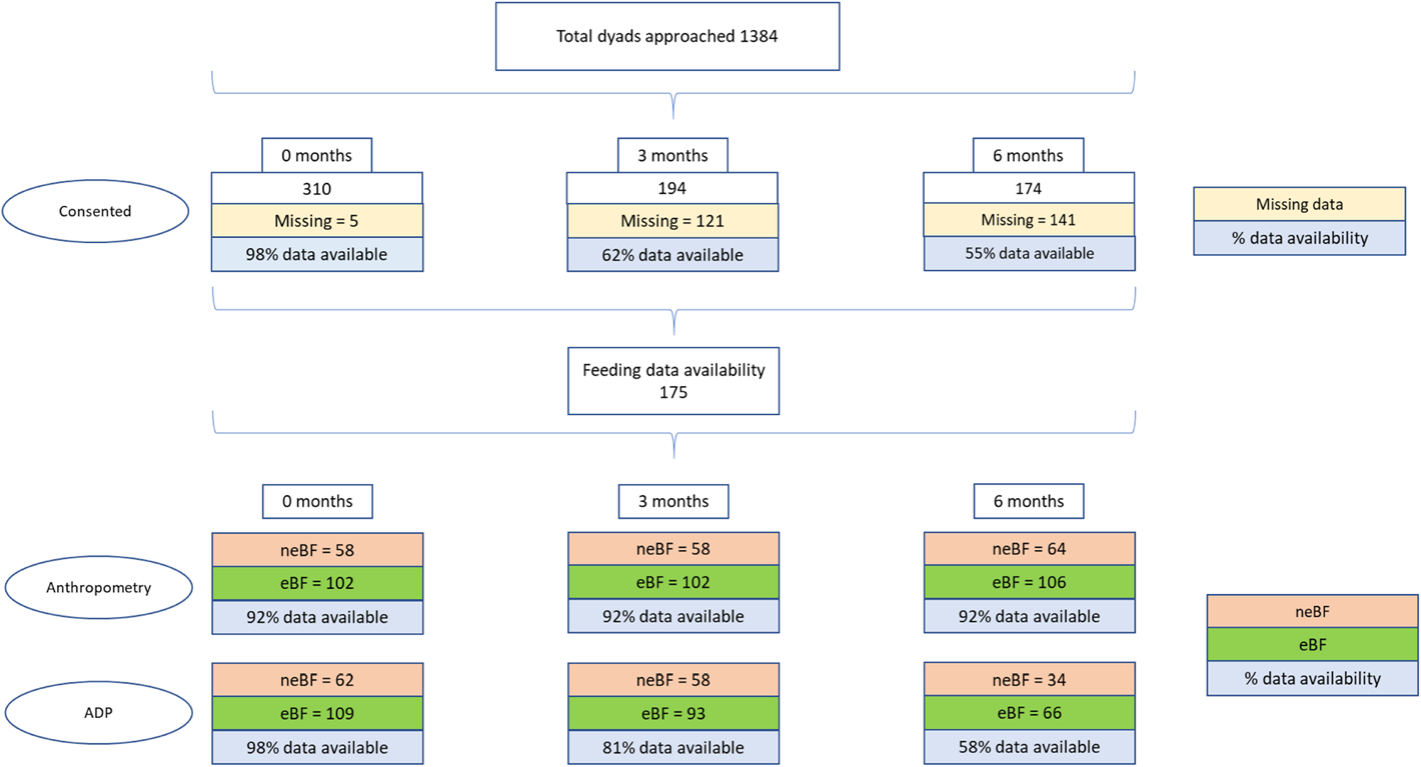
Flow diagram of study participants. Flow diagram of study participants; neBF: non-exclusive breastfeeding; eBF: exclusive breastfeeding up to 6 months

Exclusive breastfeeding was defined as the infant has received only breast milk from his/her mother, or expressed breast milk, and no other substantial amount of liquids or solids with the exception of drops or syrups consisting of vitamins, mineral supplements or medicines since birth [34, 35]. Sixty-three percent of infants were reported to have been eBF to 6 months of age. The remaining 37% were included in the neBF group, who consumed formula or a mixture of breast milk, formula, and solids. A detailed breakdown of participant numbers is shown in Fig. 1.

### Body composition and anthropometric measurements

Infant body weight, fat mass and fat-free mass were assessed via air displacement plethysmography (PEA POD, COSMED, Rome, Italy). Briefly, body weight was measured (using the integrated scale) in unclothed infants and hair flattened (with a hair cap or baby oil) prior to placing them in the automatic volume measurement capsule. Subscapular (SS) and triceps (TS) skinfolds were obtained from infants at 3 and 6 months using a calibrated skinfold caliper (Holtain Limited, Croswell, UK). Left mid-upper arm circumference (MUAC) was measured in all infants using a tape at the measured halfway mark between the acromion and olecranon processes.

Prenatal body mass index (BMI) was calculated using self-reported height and weight of participating mothers. For relevant analyses, mothers were allocated into BMI categories (underweight < 18.5; healthy weight 18.5–24.9; overweight 25.0–29.9 and obese, > 30.0 kg m^− 2^) according to World Health Organisation BMI criteria. Maternal postpartum body weight retention, length corrected fat mass and weight-for-length (WFL) in infants were enumerated as follows:

$$ {\displaystyle \begin{array}{c}\mathrm{Maternal}\ \mathrm{postpartum}\ \mathrm{weight}\ \mathrm{retention}=\mathrm{body}\ \mathrm{weight}\ \mathrm{at}\ \left[\left(3\ \mathrm{or}\ 6\ \mathrm{months}\right)-\left(\mathrm{pre}-\mathrm{pregnancy}\right)\right]\\ {}\mathrm{Infant}\ \mathrm{fat}\ \mathrm{mass}\ \mathrm{in}\mathrm{dex}=\mathrm{fat}\ \mathrm{mass}\ \left(\mathrm{kg}\right)/{\left(\mathrm{length}\ \mathrm{in}\ \mathrm{meters}\right)}^2\\ {}\mathrm{Infant}\ \mathrm{WFL}=\mathrm{weight}\ \left(\mathrm{kg}\right)/\mathrm{length}\ \left(\mathrm{m}\right)\end{array}} $$

Maternal body weight and height measures (apart from prenatal height and weight which was self-reported) were completed in duplicate (to maintain reliability) at each of the visits. Body weight was recorded to the nearest gram using a digitized scale (SECA Corp. Hamburg, Germany) and height was measured to the nearest millimetre using a stadiometer (SECA Corp. Hamburg, Germany).

### Statistical analyses

All statistical analyses were conducted using the Statistical Package for the Social Sciences (SPSS) software (SPSS Version 26, Inc., Chicago, IL, USA). Results are presented as means and standard deviations, unless specified otherwise. Cross-sectional comparisons between eBF and neBF mothers and infants were executed using independent sample t-tests. Longitudinal differences in WFL, fat mass index and BMI between eBF and neBF groups were evaluated using a repeated measures analysis of variance (ANOVA). In the event of violation of sphericity assumptions, Greenhouse–Geisser or Huynh Feldt corrections were applied as appropriate. Relationship between skinfold measures, MUAC, maternal pre-pregnancy BMI and infant fat mass content was assessed using Pearson correlation coefficient. Statistical significance was set at *p* < 0.05.

## Results

### Infant body mass and composition

**Fig. 2:**
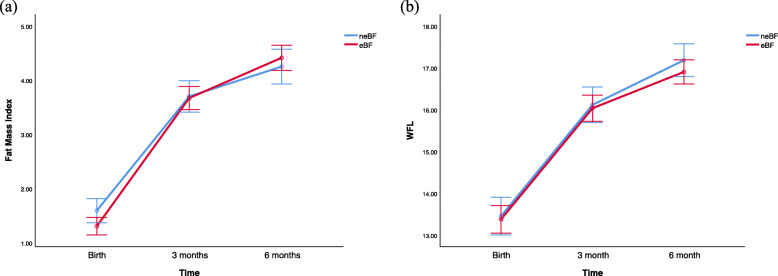
Infant fat mass index and weight for length. Infant fat mass index (**a**) and WFL (**b**) (means ±95% confidence interval) in eBF and neBF infants in the first 6 months of life; neBF: non-exclusive breastfeeding; eBF: exclusive breastfeeding up to 6 months

Body weight, fat mass, skinfold thickness and MUAC did not differ between eBF and neBF infants throughout the first 6 months of life (Table 1). Nevertheless, fat-free mass was significantly higher [*t* = 2.27, *df* = 98, *P* = 0.03, confidence interval (CI) 0.03, 0.48] in neBF infants at 6 months despite a higher mean fat-free mass in eBF infants at birth (2.89 ± 0.34 vs 3.01 ± 0.35 kg) (Table 1). Both groups of infants displayed almost identical measurements for all anthropometric and body composition parameters at 3 months (Table 1). Repeated measures analysis of variance revealed weak evidence (fat mass index lower in eBF at birth but higher at 3 months compared with neBF; Fig. 2a) for different fat mass index trajectories for eBF and nEBF infants in the first 6 months of life (ANOVA, *F* = 2.42, *df* = 1.9, *P* = 0.09). No such effect (WFL lower in eBF throughout the 6 months; Fig. 2b) was observed for WFL (ANOVA, *F* = 0.32, *df* = 1.7, *P* = 0.69). Furthermore, we observed an inversion in fat mass index levels between eBF and neBF infants from 3 to 6 months, alongside a divergence of WFL trajectory (Fig. 2a and b). There were significant correlations between SS, TS, MUAC and fat mass at 3 and 6 months, in both groups of infants (Table 5).

**Table 1:** Body weight and composition (mean ± SD) of eBF and neBF infants in the first 6 months of life

	Birth				3 months				6 months			
		neBF		eBF		neBF		eBF		neBF		eBF
	n		n		n		n		n		n	
Body weight (kg)	62	3.28 ± 0.44	109	3.35 ± 0.45	58	5.83 ± 0.69	93	5.87 ± 0.77	34	7.42 ± 0.76	66	7.24 ± 0.62
Fat mass (kg)	62	0.39 ± 0.16	109	0.35 ± 0.16	58	1.40 ± 0.37	93	1.40 ± 0.39	34	1.83 ± 0.43	66	1.90 ± 0.37
Fat-free mass (kg)	62	**2.89 ± 0.34**	109	**3.01 ± 0.35**	58	4.44 ± 0.49	93	4.48 ± 0.50	34	**5.59 ± 0.59**	66	**5.33 ± 0.50**
TS (mm)		–		–	57	10.71 ± 2.13	102	10.71 ± 1.81	64	12.78 ± 2.35	106	12.25 ± 2.43
SS (mm)		–		–	57	7.97 ± 1.68	102	7.86 ± 1.86	64	7.97 ± 1.64	106	7.67 ± 1.59
MUAC (cm)		–		–	57	12.97 ± 0.14	102	13.27 ± 0.11	64	14.50 ± 1.12	106	14.48 ± 1.22

*neBF* non-exclusive breastfeeding, *eBF* exclusive breastfeeding up to 6 months, *TS* Triceps skinfold, *SS* Subscapular skinfold, *MUAC* Mid-upper arm circumference, *SD* standard deviation

### Maternal body weight, composition, breastfeeding status and birth complications

**Fig. 3:**
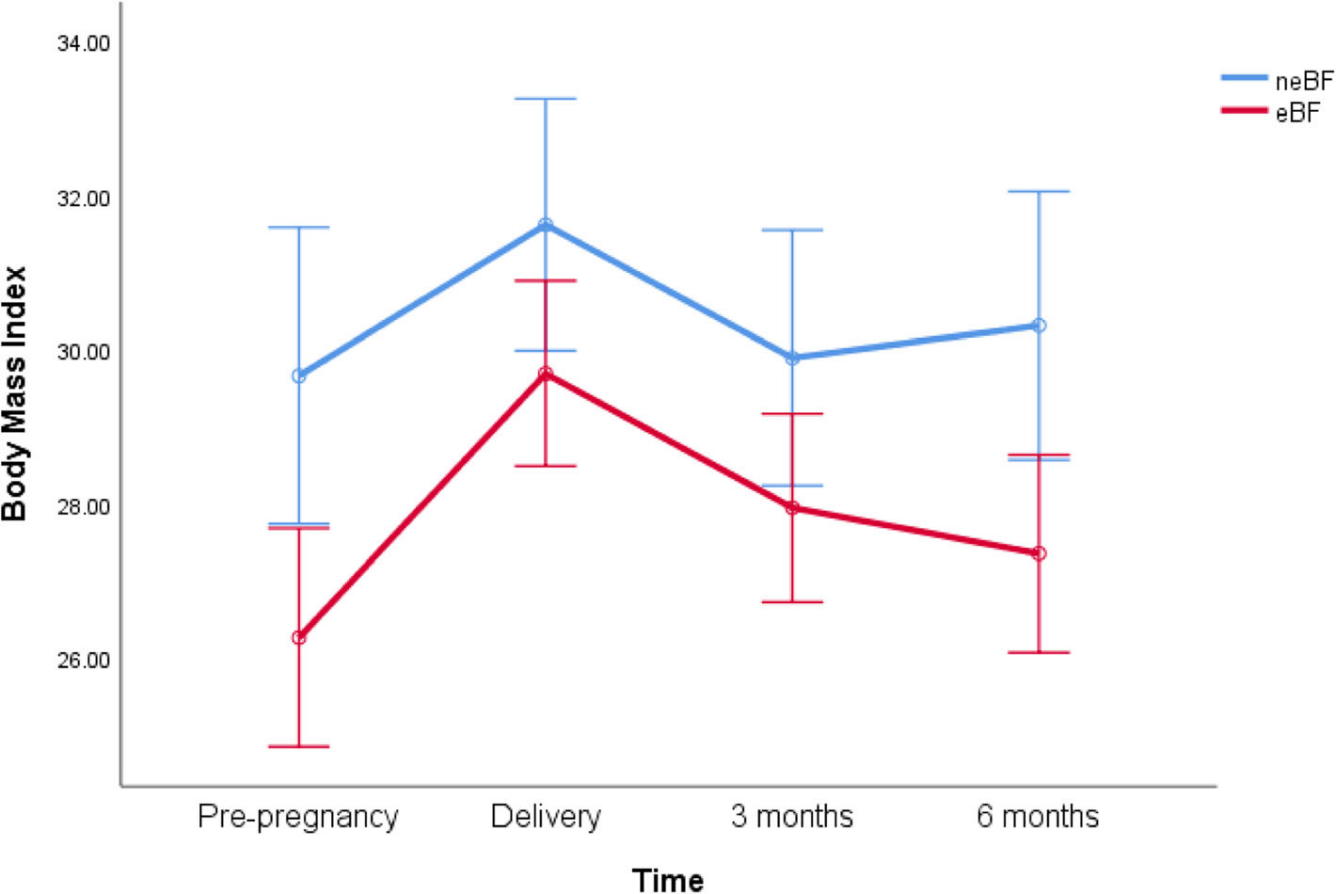
Maternal body mass index. Maternal BMI trajectory (mean ± 95% confidence interval) of eBF and neBF mothers from conception through to 6 months postpartum; neBF: non-exclusive breastfeeding; eBF: exclusive breastfeeding up to 6 months

At 3 months postpartum, the mean body weight of mothers who eBF showed weak evidence (t = 1.92, *df* = 160, *P* = 0.06, CI -0.17, 11.07) to be lower than counterparts who were neBF, and at 6 months postpartum, eBF mothers were significantly lighter (t = 3.49, *df* = 166, *P* = 0.001, CI 4.44, 15.99) (Table 2). Repeated measures analysis of variance revealed significant differences in BMI trajectories of eBF and neBF mothers through pregnancy and the first 6 months postpartum (ANOVA, *F* = 5.56, *df* = 1.8, *P* = 0.01, Fig. 3). Mothers in the neBF group, retained significantly less weight (t = − 2.75, *df* = 158, *P* = 0.02, CI -6.64, − 1.09) at 3 months (0.68 ± 11.69 vs 4.55 ± 6.08 kg) at 3 months compared with the eBF women but the amount of weight retained at 6 months was similar between groups (Table 2). Prevalence of neBF showed an increment from mothers with a normal BMI to mothers with obesity (Table 3). Further, mothers who underwent surgical or medical intervention during birth were less likely to eBF (Table 4). Maternal pre-pregnancy BMI was not significantly related to infant fat mass at 3 and 6 months, regardless of breastfeeding status (Table 5).

**Table 2:** Maternal body weight and weight retention [mean ± SD (median, inter-quartile range)] from pregnancy through to 6 months postpartum

	Pre-pregnancy		Birth		3 months		6 months	
	neBF	eBF	neBF	eBF	neBF	eBF	neBF	eBF
Body weight (kg)	**81.40 ± 22.50 (75, 24)**	**71.64 ± 16.89 (69, 18)**	**87.77 ± 20.00 (81, 22)**	**81.09 ± 15.96 (81, 20)**	82.32 ± 18.81 (77, 25)	76.86 ± 16.60 (75, 18)	**84.60 ± 20.17 (81, 26)**	**74.39 ± 16.95 (72, 20)**
Weight retention (kg)	–	–	–	–	**0.68 ± 11.69 (2, 8)**	**4.55 ± 6.08 (4, 7)**	2.51 ± 11.32 (4, 8)	2.68 ± 5.98 (3, 7)

*neBF* non-exclusive breastfeeding, *eBF* exclusive breastfeeding up to 6 months, *SD* standard deviation

**Table 3:** Level of obesity and breastfeeding status in mothers

BMI category	Breastfeeding status		Total
	neBF (***n*** = 61)	eBF (***n*** = 107)	
Underweight	0	2 (100%)	2 (1%)
Normal weight	20 (27%)	55 (73%)	75 (45%)
Overweight	20 (39%)	32 (61%)	52 (31%)
Obese	21 (54%)	18 (46%)	39 (23%)
	61 (36%)	107 (64%)	168

*neBF* non-exclusive breastfeeding, *eBF* exclusive breastfeeding up to 6 months

**Table 4:** Effects of mode of birth on breastfeeding

Mode of birth	Breastfeeding status		Total
	neBF (***n*** = 64)	eBF (***n*** = 111)	
Vaginal spontaneous	33 (32%)	69 (68%)	102 (58%)
Assisted vaginal	7 (28%)	18 (72%)	25 (15%)
Caesarean section	24 (50%)	24 (50%)	48 (27%)
	64 (37%)	111 (63%)	175

*neBF* non-exclusive breastfeeding, *eBF* exclusive breastfeeding up to 6 months

**Table 5:** Associations between infant anthropometrics (skinfolds and arm circumference), body composition (fat mass) and maternal pre-pregnancy BMI at 3 and 6 months

	3 months				6 months			
		neBF		eBF		neBF		eBF
	n		n		n		n	
SS (mm)	57	0.48*	102	0.51*	64	0.60*	106	0.41*
TS (mm)	57	0.59*	102	0.46*	64	0.36*	106	0.55*
MUAC (mm)	57	0.75*	102	0.67*	64	0.44*	106	0.63*
Pre-pregnancy BMI	59	0.21	103	0.15	60	0.01	108	0.08

*significant correlations (Pearson r), *p* < 0.05; *neBF* non-exclusive breastfeeding, *eBF* exclusive breastfeeding, *SS* subscapular skinfold, *TS* triceps skinfold, *MUAC* mid-upper arm circumference, *BMI* body mass index

## Discussion

This study illustrates the impact of eBF on mothers and infants in a selected cohort of mother-baby dyads from Tasmania, Australia. Both fat mass index and WFL had similar trajectories in the first 90 days of life, regardless of the type of feeding. In contrast, there was a deviation (from one another) in fat mass index and WFL in the second 90 days of life. Specifically, fat mass index in eBF infants, which was lower in the first 3 months, began to track higher than that of the neBF infants in the second 90 days. WFL trajectories of the two groups appear to separate from the 3-month time point onwards, after displaying identical increments in the first 90 days. Mothers with a higher BMI were less likely to breastfeed exclusively. Further, sustained breastfeeding was associated with greater postpartum maternal weight loss, confirmation of the efficacy of lactation as a weight loss strategy. Breastfeeding (and associated energy expenditure through milk production) intensifies lipolysis whereby accumulated fat tissue during pregnancy is mobilized, resulting in subsequent reductions in postpartum weight loss in mothers [36]. Medical intervention at birth also appears to be a major contributing factor that determines the pattern of breastfeeding. Although we do not have the capacity to draw on definitive conclusions in the current instance, existing empirical evidence indicate that factors such as stressful labour/birth, caesarean births, psychosocial stress/ pain due to childbirth and the consequent endocrine (e.g., oxytocin secretion) or mechanical (e.g., milk ejection reflex) changes associated with them can significantly delay lactogenesis and cause reductions in breastfeeding [37, 38].

Our observation (through fat mass index analysis) that exclusivity of breastfeeding results in increased fat mass accumulation in infants, is consistent with Gridneva et al.’s findings regarding compositional changes in the first 12 months of life [39]. As indicated in the results, overall, fat mass index was higher in the eBF infants compared with the neBF infants. On the other hand, neBF infants displayed higher fat-free mass accretion in the same period (Fig. 2). This pattern corroborates previous findings in neBF infants where significantly higher gains in fat-free mass has been observed compared with breastfed infants [40]. This suggests that the early life compositional changes that contribute to body weight gain is dependent on the feeding status of the infant. We also observed significant correlations between proxy measures of subcutaneous fat (Subscapular skinfold [SS] and Triceps skinfold [TS]) and fat mass in both groups of infants. Our findings partially concurred with existing empirical evidence that eBF is associated with preferential accretion of subcutaneous fat [41]. This notion is particularly important as localization of adipose tissue dictates its functionality and plays a major role in its contribution to the aetiology of obesity and metabolic disease [42]. Specifically, infant subcutaneous fat (between 0 and 24 months) has been shown to associated with general adiposity during adolescence and with cardio-metabolic risk factors in children as young as 6 years of age [43, 44]. The fact that we did not observe any significant associations between maternal pre pregnancy BMI and infant body composition is an anomaly as previous research has indicated an intergenerational link between maternal and infant body composition [45–47]. Fat mass and fat-free mass are sensitive indicators of the intrauterine environment, where foetal tissue developmental and regulatory planning happens [48, 49]. Methodological issues may explain at least part of the differences observed in this study. Previous Australian data that indicate associations between pre gravid BMI and neonatal body fat, had a much larger sample size (*n* = 599) compared to the current study [50].

Despite a dearth of mechanistic evidence regarding the influence of breastfeeding on infant adiposity, some research suggests that the composition of breast milk can have a significant influence on infant growth [51–53]. For instance, Gridneva et al. reported that higher total carbohydrate level in human milk is linked with greater fat-free mass whereas higher oligosaccharide content is related to greater fat mass in the first 12 months of life [54]. Other research purports a differential effect of leptin and adiponectin concentrations in breast milk on the development of infant lean and fat mass in the first year of life [55]. Further, secretory immunoglobulins are also thought to play a major role in breast milk mediated modulation of infant body composition [56].

According to the most recent Department of Health and Human Services data, only 44% of Tasmanian infants are at least partially breastfed at 6 months with entrenched socioeconomic disparities being a potential contributing factor [32, 57]. In stark contrast, 63% of the infants in our healthy cohort were eBF for the first 6 months of infancy. It is possible that the high education (i.e., ~ > 70 was university/ tertiary educated - data not reported) attainment of the current cohort of mothers may have contributed towards the higher eBF rates. Existing Australian evidence suggest that university-educated women are twice as likely to breastfeed your child for their first 6 months of their life than non-tertiary-educated women [58]. Furthermore, based on the well documented differences in growth patterns of infants from different ethnicities [59], including in the Tasmanian context, further examination to enable wider generalizability of the findings is warranted.

We observed that exclusivity of breastfeeding decreases with increasing levels of adiposity, only 46% of mothers with obesity exclusively breastfed in the first 6 months postpartum. This finding concurs with existing empirical evidence of lower rates of breastfeeding initiation and exclusivity in women with obesity [60]. Contributing factors in obesity-mediated reductions in breastfeeding include mechanical factors such as larger breasts/areolas [61, 62], suboptimal endocrine activity, [63–65] and inefficient lactogenesis [66]. Complications during labour may also affect breastfeeding patterns by delaying the onset of lactogenesis [67]. Interestingly, in the current study, mothers who experienced assisted vaginal birth or caesarean section had relatively lower levels of exclusive breastfeeding. Previous reports have linked caesarean section with reduced the likelihood/prevalence of all forms of breastfeeding from discharge to 6 months postpartum [68]. Another important observation was that mothers who maintained eBF continued to lose weight postpartum, a trend that is corroborated by previous reports [69, 70]. Specifically, between 3- and 6-months postpartum, neBF mothers showed an increment in body weight compared to their breastfeeding counterparts. It is important to note that although (overall) it appears that both neBF and eBF women gained ~ 3 kg during the study, the trajectories associated with arrival at the 6-month body weights are unique to the respective groups. Deducing from suggested mechanisms in the current literature, it could be assumed that much of this weight loss in eBF mothers is likely to be from increases in energy expenditure and distribution/mobilization of adipose tissue depots that were accumulated during pregnancy [71–73]. An ongoing challenge in this context is to differentiate between whether sustained postpartum weight loss is due to lactation per se or general/overall increments in energy expenditure, as it is likely that women who were habitually active pre-pregnancy may shed the weight gained during gestation faster than their relatively inactive counterparts [74]. Regardless, weight loss following childbirth is particularly important as excess postpartum weight retention may be associated with numerous complications including adverse metabolic conditions and entering subsequent pregnancies at successively unhealthy weight and fatness levels [75, 76].

### Limitations

Dietary self-reporting is thought to be marred by social desirability bias [77]. As such, we acknowledge there may have been limitations/ biases in some of the self-reported parameters by mothers in the current study. Nevertheless, existing literature indicate that breastfeeding initiation and duration information derived from maternal recall can be considered valid and reliable [78]. Further, volunteer bias, a well-documented impediment for participant recruitment and retention in research studies [79] may have contributed to the relatively low sample size in this instance. Reasons beyond the research team’s control such as being time poor, and relocation (to other parts of the state) were cited for the inability to continue participating in the study. In addition, due to the descriptive nature of this report, the influence of potential contributors toward endemic patterns of breastfeeding such as parity, diet, sedentary occupation, and socio-economic status, were not considered thus limiting comparability of current results with other populations.

## Conclusions

There is an urgent need for a concerted effort from all key stakeholders in maternal and infant health to promote optimal breastfeeding practices across all populations. As illustrated by the results of the current study, infants with different feeding patterns may display varying growth patterns in early life. Education and support for eBF at a population level should be accompanied by the simultaneous assessment of body composition (as opposed to widespread exclusive use of anthropometry) to obtain a more comprehensive understanding of infant growth. Accurate quantification of body composition status can provide important insights regarding qualitative and quantitative differences in specific tissue types. Logical extensions of the current research include the utilization of objective measurement approaches (e.g., deuterium dilution dose-to-mother technique) and a comprehensive evaluation of the role of different components of breast milk in shaping the longitudinal health, including body composition, of infants and young children.

## Data Availability

The datasets generated and/or analysed during the current study are not publicly available due privacy protection and ethical obligations but are available (in deidentified form) from the corresponding author on reasonable request.

## Abbreviations

BMI: Body mass index
eBF: Exclusive breastfeeding
MUAC: Mid-upper arm circumference
neBF: Non-exclusive breastfeeding
SS: Subscapular skinfold
TS: Triceps skinfold
WFL: Weight-for-length
